# Social media for public health: Reaping the benefits, mitigating the harms

**DOI:** 10.34172/hpp.2023.13

**Published:** 2023-07-10

**Authors:** Zain Jafar, Jonathan D. Quick, Heidi J. Larson, Verner Venegas-Vera, Philip Napoli, Godfrey Musuka, Tafadzwa Dzinamarira, Kolar Sridara Meena, T. Raju Kanmani, Eszter Rimányi

**Affiliations:** ^1^Trinity College of Arts and Sciences, Duke University, Durham, USA; ^2^Duke Global Health Institute, Duke University, Durham, USA; ^3^London School of Hygiene & Tropical Medicine, UK; ^4^Institute for Health Metrics & Evaluation, University of Washington, Seattle, USA; ^5^Division of Internal Medicine, Mexican Institute of Social Security, Mexico; ^6^Sanford School of Public Policy, Duke University, Durham, USA; ^7^International Initiative for Impact Evaluation (3ie), Harare, Zimbabwe; ^8^School of Health Sciences & Public Health, University of Pretoria, South Africa; ^9^Journal of Mental Health Education, Department of Mental Health Education, MIMHANS, India; ^10^Department of Psychiatric Social Work, National Institute of Mental Health and Neurosciences, India; ^11^University of North Carolina at Chapel Hill, Chapel Hill, USA

**Keywords:** Health communication, Mental health, Pandemics, Public health, Social media

## Abstract

With more than 4.26 billion social media users worldwide, social media has become a primary source of health information, exchange, and influence. As its use has rapidly expanded, social media has proven to be a "doubled-edged sword," with considerable benefits as well as notable harms. It can be used to encourage preventive behaviors, foster social connectivity for better mental health, enable health officials to deliver timely information, and connect individuals to reliable information. But social media also has contributed to public health crises by exacerbating a decline in public trust, deteriorating mental health (especially in young people), and spreading dangerous misinformation. These realities have profound implications for health professionals, social media companies, governments, and users. We discuss promising guidelines, digital safety practices, and regulations on which to build a comprehensive approach to healthy use of social media. Concerted efforts from social media companies, governments, users, public interest groups, and academia are essential to mitigate the harms and unlock the benefits of this powerful new technology.

## Introduction

 In the last two decades, social media has become increasingly prevalent in daily life. By the end of 2021, there were more than 4.26 billion social media users worldwide, a number that is expected to increase to nearly six billion by 2027.^1^ As social media has grown more ubiquitous, it has intersected with public health — for both good and bad. While becoming a powerful communication tool for health officials and public health agencies, social media has also contributed to public health crises via the spread of misinformation and the erosion of mental health. This paper will explore this duality and propose recommendations to swing the balance between social media’s advantages and drawbacks more positively.

## The growth and reach of social media for health

 Social media use has exploded in the last twenty years. The number of active monthly users is in the billions for today’s leading platforms: Facebook (2.9 billion), YouTube (2.6 billion), WhatsApp (2.0 billion), Instagram (1.5 billion), WeChat/Weixin (1.3 billion), and TikTok (1.0 billion).^2^

**Figure 1:**
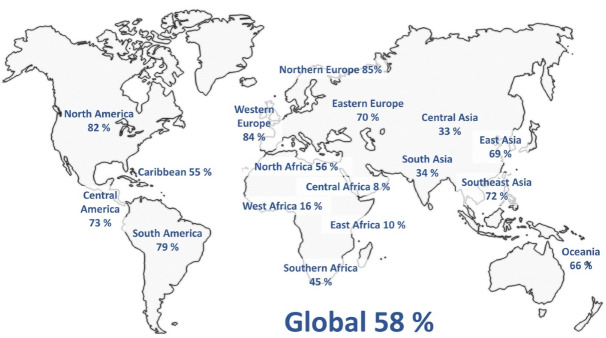
Social media users by region as a percent of total population (January 2022). Visualization: Authors. Data source: Digital 2022: Global digital overview, page 90.^4^

 In recent years social media’s growth has stabilized in high-income nations, but in low- and middle-income countries, regular use of social media has increased sharply from just 34% of people in 2013 to 53% in 2018.^3^ As of January 2022, social media usage has spread to every region of the world, with an estimated 58% of the world’s population being regular users (Figure 1).

## Benefits and harms of social media

 As global access to social media has increased, it has produced both significant benefits and substantial harms to individual and population health. While this duality of social media was highlighted during the COVID-19 pandemic,^5^ accumulating evidence demonstrates it can be seen across the spectrum of other health emergencies, health promotion and prevention, healthcare provision, and mental health (Table 1).

**Table 1:** Benefits and harms of social media for health

	**Benefits **	** Harms**
All uses	Direct access to latest information, data, evidence, and expert opinion from local, national, international sources Photos, videos, and graphics provide visual information and instruction that can be more effective and impactful SMP algorithms for searches, recommendations, and popups can facilitate access to relevant information Builds community and reduces both personal and professional isolation.	Fast spread of falsified content, fake news, manipulated information, or uninformed opinion Unsourced or fictitiously sourced information makes it hard to distinguish the reliable from unreliable data Information overload (“cognitive overload”) can lead to decline in memory, critical thinking and creativity Polarization due to SMP algorithms that funnel users into preference bubbles (“echo chambers”) Compulsive social media use (“technology addiction”) that undermines school and work performance
Health promotion and prevention	Evidence-based information and recommendations on nutrition, fitness, and other healthy habits. Access to wellness groups, chats, and other sources of peer support Assessment of pros and cons of new developments in health living and disease prevention	Aggressive marketing of tobacco products, e-cigarettes, alcohol – often targeting minors Misleading and unsafe marketing of pharmaceutical or herbal medicines, infant formula, and other products Paid influencers and sponsored content disguised as independent assessments deceive consumers
Healthcare provision	Provides direct access to current diagnosis and treatment information for users, including those in remote areas Facilitates relationships between patients and providers, and among providers and healthcare organizations Helps professional and lay groups exchange views the validity and implications of new healthcare developments	Testimonials and unverified claims encourage unproven, ineffective, costly or dangerous alternative treatments Self-diagnosis based on unreliable information or anecdotal experience can delay effective, life-saving treatment Time-consuming for providers
Pandemics and other health emergencies	Rapid local to global alerts on disease outbreaks and other health emergencies Facilitates open exchanges on benefits, costs, and alternative response options Direct access to government recommendations, requirements, protocols, and resources Identification of rumors, misinformation, conspiracy theories to adapt communication strategies	Rapid spread of misinformation and disinformation undermines trust in public health responses Active promotion of ineffective, unproven, or unsafe responses diverts attention and resources Threats, intimidation or violence against public officials or healthcare providers Panic or riots due to rumors or conspiracy theories Panic or paranoia when magnitude of earthquakes, pandemics and other natural or manmade disasters is exaggerated
Mental health	Increased positive mental health and well-being when SM interactions are encouraging and supportive Support through need-specific groups (eg, suicide prevention, Alzheimer’s support, addiction support) Access to mental health resources, professionals and other sources for help-seeking and support Increased mental health literacy and reduced stigma from information and educational campaigns Builds social connection, community preparedness and resilience for psychological and social adversities Builds connections for global perspectives on specific targeted interventions in the area of mental health	Negativity in thoughts, self-esteem or body image leading to social isolation and withdrawal Depression, anxiety disorders, self-harm and suicide, especially among girls, women, and youth Pro-suicide and suicide pact sites, message boards, chat rooms Cyberbullying and cyberstalking with resulting psychological or physical harms Anorexia nervosa, bulimia, and obesity with associated body-shaming Behavioral contagion through deadly challenge contests such as eating laundry detergent pods Disrupted sleep patterns and sleep-related disorders from excessive use of social media

Notes: SM, social media; SMP, Social media platform (eg, Facebook, YouTube, WhatsApp, Instagram, TikTok). Source: Adapted by authors from Venegas-Vera^5^, Banerjee^6^, Madziva^7^, Karim^8^, Carrion-Alvarez.^9^

###  Benefits for improved global health 

 Social media has become a significant source of public health information as it has grown more common in daily life. This was seen during the COVID-19 pandemic, according to a Pew Research study that found almost half of Americans have been getting a lot or some information regarding COVID-19 vaccines from social media.^10^ By acting as an information resource, social media has often encouraged preventive behaviors. A study conducted in Bangladesh, for example, found that social media users were about three times more likely than non-users to follow health rules established in response to the pandemic.^11^ And a study from NYU School of Global Health found that informal sources like social media had the greatest potential to sway individuals towards preventive behaviors against the virus.^12^

 Social media, therefore, offers a new tool for health officials to improve health literacy, raise awareness, and reach a larger audience with timely truths ahead of fatal fictions.^13^ This was the case with Ebola^14^ as well as with COVID-19 in West Africa, for which a study that found the number of social media accounts an individual owned was a significant predictor of their awareness of methods to stop the spread of the COVID-19 virus.^15^ Social media is particularly effective during times of crisis because it allows valuable information to be spread widely and rapidly,^5^ enhancing awareness and prevention strategies among the public.^6^ A 2020 systematic review highlighted the growing importance of social media in rapid detection of health-related rumors during outbreaks.^16^

 Positive mental health benefits also have been associated with social media usage. One study from 2019 found that routine social media use was positively related to mental health, self-rated health, social well-being, and resilience.^17^ The authors of this study believe social media can be beneficial by offering meaningful social interactions and opportunities to maintain existing social networks. A survey of American teens echoed this sentiment, with 81% saying social media makes them feel more connected to their friends’ lives and 68% saying social media makes them feel that they have a support network when they encounter hardships.^18^ Social media can also help to decrease feelings of loneliness, especially among those with underlying mental health issues.^19^ Social media offers new opportunities to disseminate mental health information, connect individuals to mental health services, and create support networks between individuals and their caregivers.^20^

###  Unintended harms 

 Despite significant health benefits, social media has been associated with substantial negative health impacts as well. While preventive behaviors may have been encouraged via social media usage, vaccine hesitancy has also been fueled by such sites,^21^ largely through unrestrained forms of misinformation that abound on these platforms.^22^ One study attempting to establish a causal relationship between exposure to social media misinformation and vaccine hesitancy found a 6.4-percentage point decrease in the number of individuals who would “definitely” receive a COVID-19 vaccine relative to individuals who did not receive any misinformation exposure in the United States, and a 6.2-percentage point decrease in the United Kingdom.^23^ A review of the 100 most popular YouTube videos on autism spectrum disorder found that all but one were posted by non-professionals and 16% promoted the disproven vaccine-autism link.^24^ Another study found a 2-percentage drop in mean vaccination coverage each year with every 1-point increase on a 5-point disinformation scale.^25^ In Japan, social media was weaponized by anti-HPV vaccine groups to spread misinformation so drastically that it pushed the government to redact its proactive recommendation for receiving the HPV vaccine. This policy change is estimated to have resulted in about 25 000 additional cervical cancer cases and 5000 to 5700 preventable deaths.^26^

 Another consequence of this unbridled misinformation is the use of social media to attack scientists and healthcare providers. One survey found that at least a quarter of US physicians have experienced attacks or harassment on social media during the COVID-19 era.^27^ Health officials and doctors frequently are the target of posts containing misinformation that undermines their authority. They often face severe vitriol when discussing research or issuing recommendations on social media, which may discourage future communication of crucial information.

 A wealth of evidence has found that social media is associated with significant adverse mental health effects – especially among young adults. A review of social media and adolescent well-being in the Global South showed that, despite some benefits, social media brings numerous harms to mental health.^28^ Another study concluded that social media use is a common driver of mental health issues among youth in India.^29^ A study from Cape Town, South Africa found associations between social media use and both sleep difficulties and depressive symptoms.^30^ Studies from Iran observed that individuals who did not use social networks like Instagram and Facebook had significantly lower levels of anxiety and depression^31^ and that social media use by adolescents was correlated with lower life satisfaction as well as lower family social capital.^32^

 A *Lancet* report from the UK Millennium Cohort Study established a correlation between greater social media use and online harassment, poor sleep, low self-esteem, and poor body image, which ultimately were related to higher depressive symptom scores among teen users.^33^ These findings were further supported by a recently published randomized controlled trial, which found significant differences in well-being, anxiety, and depression between groups instructed to continue using social media (Facebook, Twitter, Instagram, and Tik Tok) for a week versus those instructed to cease social media usage for a week.^34^

 Social media companies have reached similar conclusions internally. In the last four years, Facebook has repeatedly determined that Instagram — used by roughly three-quarters of teens in the United States^35^ — is harmful for many of these young users, and that teenage girls are particularly affected.^36^ Approximately 40% of Instagram’s global users are below the age of 24.^37^

 A trove of released company documents called the Facebook Papers revealed internal research that found one-third of teenage girls with body image issues have these issues made worse when they use Instagram, which is owned by Facebook. The company’s own research also reported that “teens blame Instagram for increases in the rate of anxiety and depression.” Facebook studies found that 13% of suicidal teenagers in Britain and 6% in the United States attributed their suicidal ideation to Instagram. Finally, internal slide decks featured in the dossier showed that significant percentages of teen users reported that negative feelings — such as not feeling good enough (24% in the US, 29% in the UK), feeling alone or lonely (21% in the US, 18% in the UK), not feeling attractive (41% in the US and 43% in the UK), and feeling like they have to create the perfect image (39% in the US and 51% in the UK) — are traceable to Instagram.^36^

## How does social media cause harm?

 Social media is not inherently good or bad, but the roots of its harms may lie in the algorithms and decisions made by social media companies. Facebook’s algorithm, for example, is based on “engagement,” meaning that posts which get more likes and comments are amplified. These posts tend to be controversial or strongly resonate with users.^38^ Consequently, the algorithm has polarized users and generated networks of like-minded people, according to a study from the University of Denmark.^39^ The effect of such echo-chambers was documented by this study, which found that a person’s vaccine stance resulted in seeing “highly dissimilar sources of vaccine information.” Because of polarization based on engagement, vaccine-hesitant or anti-vaccine individuals are likely to see content that confirms their biases, causing them to engage in risky, counterproductive behaviors.

 Social media’s harm to health also stems from its addictive nature. Addiction is at the core of many social media companies’ business models: these apps feature hallmark ingredients of behavioral addictions, and social media algorithms capitalize on neurochemical responses to keep users returning.^40^ Some teens, aware of Instagram’s harms, have tried to distance themselves from the app, but researchers have found the app has triggered addiction.^38^ A recent report found that 31% of social media use is caused by self-control problems,^41^ indicating a real connection between addiction and social media use. The addictive nature of social media algorithms leaves young brains continually vulnerable.

 The risks of social media have been intensified by social media companies’ deliberate negligence in the face of such harms, as revealed by an October 2020 piece published in *The New Yorker*. The exposé suggests that Facebook’s repeated inaction is a byproduct of a business model that prioritizes engagement and that its laissez-faire approach towards divisive content and hate speech is more lucrative.^42^ This apathy and its impact on public health were displayed in Facebook’s response to misinformation during the pandemic. According to the Facebook Papers, when Facebook’s employees devised strategies to suppress posts featuring misinformation and promote factual content from public health agencies during the vaccine rollout, company leadership balked at these proposals.^43^ The Facebook Papers also show that when the company initiated a pilot program that reduced debunked claims by 12% and boosted content from public health agencies by 8%, the company hesitated on wide-scale implementation. Critics have pointed to a fear of decreased profits due to lower engagement as the reason for this decision.

## Strategies to promote the benefits and reduce the harms of social media

 The many benefits of social media outlined in Table 1 are largely driven by the actions of its users engaging through the platforms provided by social media companies. The harms of social media, which are not inevitable, arise primarily from a combination of the social media business model and the behavior of its users. Strategies to maximize the benefits and minimize the harms will require action by social media companies, governments, users, and civil society.

###  Social media companies

 Despite an overarching pattern of initial inaction, social media companies like Facebook have taken some steps to mitigate harm. In 2020, Facebook deployed a “viral circuit breaker” — a tool that does not take down a controversial post but instead limits its amplification. Applauded by advocates, this new feature deviated from the company’s previous strategy of issuing fact-checks days after baseless posts became widely circulated. Facebook also released a new feature in July 2022 that allows users to view posts chronologically, rather than in an algorithm-dictated feed.^44^ These are two promising measures critical to mitigating social media’s negative effects. Viral circuit breakers and chronological feeds offer a chance to disrupt algorithms and lessen the viral spread of misinformation, risks of social media addiction, and problematic usage of social media.^45^

 The flag-and-fact-check feature is another viable tool social media companies could deploy more widely to reduce the spread of misinformation. This tool denotes posts with misinformation and directs users to credible sources for accurate information. During the pandemic, many social media sites, including Facebook, Instagram, and Twitter, implemented this system, which has made people less inclined to share misinformation.^46^ One study found that flagging misleading tweets prompted users to lower their opinion on the usefulness, trustworthiness, helpfulness, and accuracy of these tweets.^47^ Such mechanisms need to be consistently in place and not come and go with the ebb and flow of world events.

 Research also indicates that changing health behaviors is more effective when social media users are exposed to accurate information before they encounter misinformation — an approach referred to as “pre-bunking.”^48^ Conversely, behavior change becomes more challenging when fact-checks are displayed after a user has been exposed to misinformation. One American study demonstrated that users’ vaccination intentions only improved if they were shown truths before they encountered misinformation.^49^ Recent research from Cambridge University and Google also shows that pre-bunking has a range of positive effects on how social media users process misinformation.^50^ Therefore, social media companies should consider proactively incorporating informative posts into users’ feeds, too.

###  Governments

 Maximizing social media’s benefits and mitigating its adverse impacts will also require systemic changes in how these platforms operate, which governments must enforce. Some experts recommend that the US government raise the minimum age for users in the Children’s Online Privacy Protection Act with more stringent age verification.^51^ Others advise breaking up social media conglomerates like Facebook, as this would make more user-friendly sites and protections against misinformation a source of competitive advantage, according to one comprehensive overview of experts’ recommendations.^52^

 Some pundits have also called for criminal penalties for executives, policy reforms, and the creation of regulatory administrations.^52^ Criminal liability would address the culture of impunity at companies like Facebook that has led these problems to spiral. Amendments to Section 230 — a landmark American law meant to uphold free speech that ultimately protects tech companies from the legal consequences of harmful content^53^ — could include requirements for social media companies, many of which are based in the US, to be held responsible for the harmful effects of amplifying dangerous content. Regulatory bodies exist for other powerful industries but are scarce for social media. Being a relatively new technology, many aspects of these platforms fall between the cracks of the jurisdiction ascribed to existing regulatory agencies such as the U.S. Federal Trade Commission and the U.S. Federal Communications Commission.

 The European Union has become a pioneer in social media regulation, to which other countries might look. The EU passed both the Digital Services Act and the Digital Markets Act in early 2022. The first of these two laws may be especially consequential to public health, as it requires social media companies to more robustly police their platforms for inflammatory posts and disinformation, hone their unrestrained algorithms, and assume greater responsibility for social media’s harms.^54^

###  Users and civil society 

 The business model, algorithms, content moderation practices, and other actions of social media companies clearly play a major role in determining balance between the beneficial and the harmful effects of social media. However, there is much that users themselves – supported by civil society – can do to tilt the balance to the positive side.

 Professional associations, consumer groups, religious bodies, and others have developed guidelines for effective, safe, and ethical use of social media. Recognizing the value of social media for health professionals, students, and patients, the World Medical Association has provided practical guidance on professional and ethical use of social media.^55^ U.S. nursing schools are beginning to introduce social media use and cybercivility guidelines.^56^ A 2019 review of social media guidelines for health professionals and faculty members from five countries concludes with practical advice on training in social media skills.^57^

 A 2022 literature review identifies ways in which users can “rewire their social media habits” for their wellbeing.^58^ For example, users can choose to use sites with explicit content guidelines and boycott those without them. They can flag harmful and misleading posts while sharing informative content related to health.

 Civil society organizations also play a central role by promoting healthy social media use and pressuring lawmakers and social media companies. Concerted movements and organizations like the Organization for Social Media Safety and the Council for Responsible Social Media institutions help to raise awareness of social media’s harms, rally public support, and instigate meaningful change.

 The Center for Human Technology (http://www.humanetech.com), founded by Google’s former Design Ethicist to “align technology with humanity’s best interests”, provides a wealth of resources on wise use of social media for individuals, organizations and policymakers. The Center’s 2020 docudrama, *The Social Dilemma* – with an estimated 100 million worldwide views – and its associated solutions and resource materials provide specific insights and tools for healthy use of this technology.^59^

## Conclusion

 Social media is a “double-edged sword” that both greatly benefits and can seriously harm individual and collective health.^13^ Social media is a tool that can encourage preventive behaviors and raise awareness rapidly, but it also can be leveraged to spread misinformation and disparage healthcare workers. Social media has been shown to have negative associations with mental health, while simultaneously providing resources and support for positive mental health.

 These realities have profound implications for social media companies, governments, health professionals, users, and civil society. The strategies to promote the benefits and reduce the harms of social media described in this perspective provide promising approaches by which these stakeholders increase the benefits and reduce the risks of social media.

 A significant issue, however, is the dearth of evidence surrounding the effectiveness of such interventions.^60^ The evaluations that do exist are predominantly from Western nations — even though nine of the ten countries with the highest number of Facebook users are in the Global South.^61^ Great investment and data-sharing is needed to generate studies on the impact of these and other interventions. One initiative that has been recently launched in this direction is the Alliance for Advancing Health Online (AAHO). Concrete indications of what works and what does not will allow for more effective, evidence-based interventions.

 Under Hippocrates’ principle of “first do no harm,” it is incumbent on the global health community, national authorities, users, and social media companies themselves to take steps to continue to mitigate social media’s harms. Social media companies need to reform policies and implement stronger control measures while strengthening the positive assets of their technology. Concurrently, governments, health organizations, NGOs, and academic and research institutions must work together for a common goal of improving mental and physical health.

## Acknowledgments

 The researchers thank Michael Penn for providing valuable insight on the benefits of social media and support throughout the duration of the project. We are also appreciative of the editorial advice provided by Karl Leif Bates.

## Ethical Approval

 Not applicable.

## Competing Interests

 In the past 36 months, HLJ has received a GSK research grant in conjunction with UK NIHR, a Merck research grant in conjunction with the MacArthur Foundation, and a J&J research grant in conjunction with the Gates Foundation.

## Funding

 No designated funding was received for the preparation of this paper.
